# Regulating the blink: Cognitive reappraisal modulates attention

**DOI:** 10.3389/fpsyg.2014.00143

**Published:** 2014-02-21

**Authors:** Ruth Adam, Sandra Schönfelder, Johanna Forneck, Michèle Wessa

**Affiliations:** ^1^Section for Experimental Psychopathology and Neuroimaging, Department of General Psychiatry, Center for Psychosocial Medicine, University of HeidelbergHeidelberg, Germany; ^2^Department of Clinical Psychology and Neuropsychology, Institute for Psychology, Johannes Gutenberg-University of MainzMainz, Germany; ^3^Research Centre Translational Neuroscience–Neuroimaging Centre, Johannes Gutenberg-University of MainzMainz, Germany

**Keywords:** affect, attentional blink, emotion, regulation, top-down

## Abstract

Our brain is unable to fully process all the sensory signals we encounter. Attention is the process that helps selecting input from all available information for detailed processing and it is largely influenced by the affective value of the stimuli. This study examined if attentional bias toward emotional stimuli can be modulated by cognitively changing their emotional value. Participants were presented with negative and neutral images from four different scene-categories depicting humans (“Reading”, “Working”, “Crying” and “Violence”). Using cognitive reappraisal subjects decreased and increased the negativity of one negative (e.g., “Crying”) and one neutral (e.g., “Reading”) category respectively, whereas they only had to watch the other two categories (e.g., “Working” and “Violence”) without changing their feelings. Subsequently, subjects performed the attentional blink paradigm. Two targets were embedded in a stream of distractors, with the previously seen human pictures serving as the first target (T1) and rotated landmark/landscape images as the second (T2). Subjects then reported T1 visibility and the orientation of T2. We investigated if the detection accuracy of T2 is influenced by the change of the emotional value of T1 due to the reappraisal manipulation. Indeed, T2 detection rate was higher when T2 was preceded by a negative image that was only viewed compared to negative images that were reappraised to be neutral. Thus, more resources were captured by images that have been reappraised before, i.e., their negativity has been reduced. This modulatory effect of reappraisal on attention was not found for neutral images. Possibly upon re-exposure to negative stimuli subjects had to recall the previously performed affective change. In this case resources may be allocated to maintain the reappraised value and therefore hinder the detection of a temporally close target. Complimentary self-reported ratings support the reappraisal manipulation of negative images.

## INTRODUCTION

We are constantly surrounded with signals from various sensory modalities, yet our neuronal system is capacity-limited and is unable to process all of the available information (Desimone and Duncan, 1995; Marois and Ivanoff, 2005). Attention is a process that guides us in selecting specific input from all available information for further, more detailed, processing (Talsma et al., 2010). Experimental instructions or information stored in working memory (Wolfe et al., 2003; Gazzaley et al., 2005; Soto et al., 2005; Gilbert and Li, 2013) can guide our attention via top-down attention modulation. For example, neuronal activations in primary visual cortex (Li et al., 2004) and neuronal selectivity in the prefrontal cortex (Cromer et al., 2010) change according to the experimental task, even when the visual stimuli are kept unchanged. On the other hand, it is well known that salient stimuli, such as emotional images, capture our attention in a bottom-up manner (Desimone and Duncan, 1995; Egeth and Yantis, 1997; öhman et al., 2001; Buschman and Miller, 2007; Zhang et al., 2012). One experimental paradigm that is widely used to study capacity limitation is the visual attentional blink (AB; Raymond et al., 1992). In the AB paradigm, subjects often miss the second (T2) of two visual targets (T1 and T2) presented in rapid serial visual presentation (RSVP) of distractors, if the two targets are presented within a short time window of 200–500 ms (Shapiro et al., 1997; Dux and Marois, 2009). The failure to report T2, also known as blinking, is explained by several different mechanisms (for review see, Dux and Marois, 2009; Martens and Wyble, 2010). According to bottleneck models, the AB occurs due to capacity limitation at the stage of stimulus-encoding into working memory (Chun and Potter, 1995; Jolicoeur, 1998; Dell’acqua et al., 2009). Alternatively, other models suggest that the limited T2 processing is due to insufficient allocation of attentional resources to that target, as governed by dynamic attentional control mechanisms (Di Lollo et al., 2005; Nieuwenstein, 2006; Olivers et al., 2007). The AB paradigm has been used to study the effect of the emotional value of stimuli on attention. Emotional stimuli can either increase or decrease T2 detection rate, depending on their location in the RSVP. When T2 is an arousing word, performance in the AB task increases (Anderson and Phelps, 2001; Keil and Ihssen, 2004; Anderson, 2005; Schwabe et al., 2011). On the other hand, emotional T1s cause a decrease in T2 detection accuracy (Schwabe et al., 2011). Similarly, in a paradigm known as the emotional AB, when subjects are asked to detect only one target in a RSVP and one of the distractors preceding T2 is an arousing stimuli, detection of the neutral T2 target is largely impaired (Arnell et al., 2007; Most et al., 2007).

It is not surprising that emotional stimuli capture our attention. They might indicate danger and thus require immediate response, facilitating survival (Hansen and Hansen, 1988; öhman et al., 2001; Keil and Ihssen, 2004; Schupp et al., 2004; Anderson, 2005; Schimmack and Derryberry, 2005). Although attending to emotion-eliciting stimuli is adaptive, dealing with emotions and the ability to regulate them is necessary for mental health and general well-being (Gross and John, 2003; Eftekhari et al., 2009; McRae et al., 2012b; Boden et al., 2013; D’Avanzato et al., 2013). Emotion regulation (ER) strategies are ways to control and change emotional responses to internal processes or external stimuli. Cognitive ER strategies range from attentional control processes in which attention is directed away from the stimulus to cognitive change of the affective situation (Gross, 1998; Ochsner and Gross, 2005). Reappraisal is a prominent ER strategy representing cognitive change and it mainly involves reinterpretation of the event, in either a situation- or self-focused manner (Lazarus and Alfert, 1964; Ochsner et al., 2004). Self-focused reappraisal relates to the modification of the relation and relevance of oneself to the situation (distancing yourself from the situation, becoming a detached observer), whereas the situation-focused approach deals with reinterpreting the situation or changing its outcome. Behaviorally, a recent meta-analysis pointed out that reappraisal is one of the most effective strategies in modulating affective responses (Webb et al., 2012), with the two reappraisal strategies having a similar behavioral effect (Ochsner et al., 2004; Webb et al., 2012). Reappraisal successfully modulates behavioral responses to emotional stimuli (Foti and Hajcak, 2008; Kanske et al., 2011; McRae et al., 2012a; Schönfelder et al., 2013), as well as physiological measurements such as heart rate (Hofmann et al., 2009) and skin conductance level (McRae et al., 2012a). Neuronally, reappraisal reduces the magnitude of the late positive potential (LPP) which is a signature of heightened emotional processing in electroencephalography (EEG) studies (Foti and Hajcak, 2008; Moser et al., 2009; Thiruchselvam et al., 2011; Paul et al., 2013; Schönfelder et al., 2013; for review see Hajcak et al., 2010) and by decreased amygdala activation as shown by functional magnetic resonance imaging (fMRI) measurements (McRae et al., 2010; Kanske et al., 2011; Golkar et al., 2012; for review see Kalisch, 2009; Ochsner et al., 2012). Moreover, few studies have observed lasting effects of reappraisal (Walter et al., 2009; Macnamara et al., 2011; Thiruchselvam et al., 2011). Those studies have shown altered behavioral affective ratings as well as neuronal signatures when subjects observed negative images that were previously reappraised.

**FIGURE 1:**
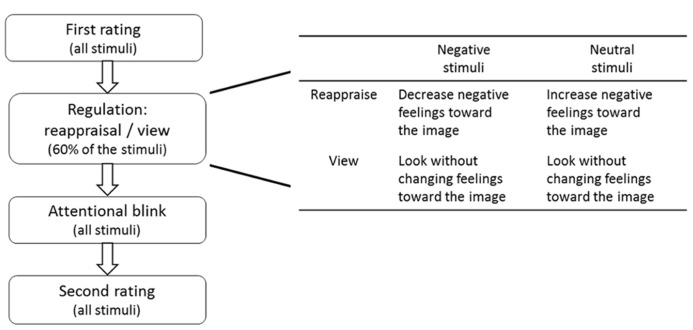
**Schematics of the experimental procedure and the conditions in the regulation part**.

Although both ER and attentional mechanisms are widely studied, the interaction between those two processes is largely unknown. The cognitive reappraisal effect is largely attributed to conscious, top-down and dynamic cognitive change of the affective situation (Urry, 2010; Gyurak et al., 2011; Paret et al., 2011; Thiruchselvam et al., 2011; McRae et al., 2012c). It brings the emotions into conscious attention, has long lasting effect on memory, influences performance under conflicting situations such as the Stroop task (Dillon et al., 2007; Moser et al., 2010; Kim and Hamann, 2012; Ortner and de Koning, 2013) and neuronally affects frontoparietal attention networks (Wessing et al., 2013). However, can reappraisal modulate the effect emotion has on attention? The current study aimed to examine if and how reappraisal affects selective attention. In an AB paradigm subjects had to detect pictures depicting humans (T1) and report the rotation of a landmark/landscape image (T2) embedded in a stream of distractors. The T1 human images were taken from two neutral (“Reading” and “Working”) and two negative (“Crying” and “Injury”) scene-categories. In a regulation stage preceding the AB task, subjects were asked to increase their negative emotions toward pictures from one neutral category (e.g., “Reading”), decrease their negative emotions toward images from one negative category (e.g., “Crying”) and passively view the images from the other two categories (e.g., “Working” and “Injury”) without changing their emotions (**Figure 1**). Therefore, one negative and one neutral category kept their original affective value while the affective values of the other two categories were changed due to the reappraisal instruction. Since we manipulated the scene/situation depicted in the pictures and in order to limit variability in the regulation-strategy employed, subjects were instructed to use situation-focused reappraisal. In the current design only 60% of T1 images from each scene-category appeared in the regulation stage while all were presented in the AB task. This enabled us to investigate the generalization of the reappraisal effect, i.e., whether applying reappraisal strategy to stimuli of a certain category (e.g., “Crying”) could produce a similar (emotion-reducing) effect on stimuli of the same scene-category that were not reappraised before.

We hypothesized that T2 detection rate would depend on the affective value of T1 images *after *the cognitive change. This can be explained by two possible mechanisms. First, reappraisal may cause a permanent, automatic change to the affective value. A second possibility is that upon presentation individuals first consider the original affective value of the image and only subsequently retrieve its new emotional meaning from memory. This retrieval process consumes resources and therefore attenuates performance in the AB task. For neutral images, the same behavioral effect can be expected in the case of both mechanisms. T2 accuracy should be reduced following reappraised neutral images irrespective of the underlying explanation. In the first case, negatively reappraised neutral T1 images are thereafter perceived as more negative which consequently reduces T2 detection rate; in the second case the retrieval process itself takes time and resources and therefore leads to the same result. On the other hand, observing the AB performance pattern for negative T1 that were either passively viewed or reappraised to be neutral enabled us to discriminate between the two mechanisms. In accordance with the first mechanism (Arnell et al., 2007; Most et al., 2007), we would expect passively viewed negative images (i.e., no change in affective value) to capture more resources and thus attenuate AB performance compared to negative images that were reappraised as neutral. On the other hand, according to the second mechanism, the opposite effect is hypothesized (Ortner et al., 2013). An increased number of blinks is expected for negative images that were previously reappraised as neutral since the retrieval process of the new reappraised interpretation captures resources that are needed for T2 processing.

## MATERIALS AND METHODS

### PARTICIPANTS AND DIAGNOSTIC ASSESSMENT

30 subjects participated in this study and were either rewarded with monetary gain or course credits. Five subjects had to be excluded from the study due to incompletion of the experiment (one subject), interruption to the experiment (one subject), reported confusion between the response keys (two subjects), and poor accuracy at the main task (performances below the mean - 2STD, one subject) leaving 25 subjects in the analysis (12 females, mean age 24.7 years, range 20–37 years, 23 right-handed). All participants had normal or corrected to normal vision, were healthy and reported no history of mental disorders or head injuries as confirmed by an exhaustive phone screening interview. This screening procedure reviewed the following exclusion criteria: visual or hearing impairments, a lifetime history of head injury with loss of consciousness, brain damage or surgery, neurological illness or mental disorder, alcohol or drug abuse, and regular medication use (excluding oral contraceptives). Participants gave written informed consent prior to the study, which was approved by the Ethics Committee of the Medical Faculty Heidelberg, University of Heidelberg.

### STIMULI

Visual stimuli were grayscale pictures centered on a black background (16 cm × 12 cm, visual angle 16.3° × 12.2°). Images were selected from the International Affective Picture System (IAPS; Lang et al., 2008), the Emotional Picture Set (EmoPicS; Wessa et al., 2010), the Nencki Affective Picture System (NAPS; Marchewka et al., 2013) and the Internet.

#### T1 images

60 images depicting human, all taken from validated picture sets (16 IAPS, 36 EmoPicS, 8 NAPS)^1^, were subjected to our experimental manipulation and were rated, reappraised or passively viewed, and served as the first target (T1) in the AB paradigm. Stimuli were divided into two neutral (“Reading” and “Working”) and two negative (“Crying” and “Injury”) scene-categories, with 15 images each. By design, the neutral and negative conditions significantly differ in their normative valence and arousal values [multivariate analysis of variance (MANOVA) with valence and arousal as dependent variables, valence: *F*(1,58) = 575.96, *p *< 0.001; arousal: *F*(1,58) = 238.51, *p *< 0.001]. Normative valence and arousal ratings did not significantly differ between the two neutral and the two negative conditions [“Crying” vs. “Injury” valence: *t*(28) = 1.01, *p *= 0.322; “Crying” vs. “Injury” arousal: *t*(28) = -1.02, *p *= 0.316; “Reading” vs. “Working” valence: *t*(28) = 0.84, *p *= 0.407; “Reading” vs. “Working” arousal: *t*(28) = -1.35, *p *= 0.186; **Table 1**]. Six stimulus-pairs were created for every category by pairing similar pictures (e.g., in the “Reading” category a picture showing an elderly man reading a newspaper was paired with that of a young man reading a magazine; in the “Crying” category a picture of a sobbing man behind smoky ruins was matched with that of a crying woman behind a smoky living complex). One image from every pair and additional three pictures were shown in the reappraisal part, amounting to a total of nine pictures per scene-category in this experimental phase (see Design and procedure).

**Table 1:** Mean valence and arousal values (and SEM) for the T1 images from the four scene-categories.

	Normative values		Sample ratings-first session	
	Valence	Arousal	Valence	Arousal
**Neutral categories**
Reading	5.25 (0.12)	3.02 (0.16)	5.91 (0.13)	1.41 (0.09)
Working	5.09 (0.14)	3.30 (0.13)	5.38 (0.09)	1.51 (0.11)
**Negative categories**
Crying	2.69 (0.07)	5.58 (0.20)	3.46 (0.13)	3.39 (0.31)
Injury	2.58 (0.08)	5.84(0.16)	3.20 (0.15)	4.26 (0.37)

*Normative values and values from the first rating session are presented.
*

All stimuli were resized to 800 × 600 pixels. Some images were cropped and resized to either remove black-pixel borders present in the original image, or to optimize the size of humans in the final image. Additionally, to make sure all subjects were paying attention to the same human depiction, ambiguous/blurry depictions of humans were removed. In order to avoid potential effect of low-level visual feature on attention and thus bias the AB performance, color information was removed and the contrast of the grayscale images was adjusted. As a result the four categories did not significantly differ in terms of luminance [*F*(3,56) = 0.21, *p *= 0.887] or contrast [*F*(3,56) = 0.41, *p *= 0.745] values. Those changes are not supposed to alter the affective perception of the pictures (see Bradley et al., 2003, 2011; for showing that perceptual differences cannot explain emotional modulation as well as brain activation evoked by emotional stimuli). Nonetheless, affective ratings were collected from each participant.

#### T2 and distractor images

The AB task included additionally 120 architectural and landscape images taken from the Internet, out of which 60 were used as distractor stimuli and 60 as T2 stimuli (30 images each rotated 90° clockwise and anticlockwise, amounting to 60 stimuli). The luminance and contrast values of T2 and distractor images were adjusted to the overall T1 values, giving similar values across T1, T2 and the distractor images [luminance: *F*(2,177) = 1.07, *p *= 0.344; contrast: *F*(2,177) = 1.97, *p *= 0.143].

#### Training images

Additional 31 negative and neutral pictures depicting humans were used for rating, reappraisal and AB trainings.

### DESIGN AND PROCEDURE

The experiment included four parts in the following order: (1) first rating of valence and arousal, (2) regulation phase that included cognitive reappraisal and passive view conditions, (3) AB task, and (4) second rating of valence and arousal. In addition, the subjects filled in several questionnaires at the end of the experiment (**Figure 1**).

The 2 × 2 × 2 factorial design manipulated the (1) initial (normative) emotional value of the category (two: negative, neutral), (2) regulation-strategy applied on the scene-category (two: reappraise, passive view), (3) novelty (two: images seen at the regulation part, images not seen at the regulation part). T2 detection accuracy in the AB task as well as valence and arousal ratings served as dependent measures.

#### Rating tasks

After a 3000 ms picture presentation, participants rated their current emotional-state to T1 human images as indicated by valence (from pleasant to unpleasant) and arousal (from relaxed to stimulated) ratings on a 9-point scale using the Self-Assessment Manikin scales (SAM; Bradley and Lang, 1994; maximal duration: 4000 ms per rating). The order of those two questions was counter-balanced among participants. Pictures were interleaved with 2000 ms fixation cross. The second rating task was aimed at evaluating the shift in affective response to the images due to the reappraisal manipulation. In this stage participants rated in addition the T2 images in their upright orientation. The first and second rating sessions lasted 13 min and 26 min, respectively (excluding a short break in the middle). Before the first rating session subjects completed three training trials.

#### Regulation task

Subjects encountered two strategies: (1) *reappraise* (indicated by the German word VERÄNDERN and an arrow indicating the direction of the change) and (2) *passive view* (indicated by the German word ANSCHAUEN). In the first condition subjects were asked to change their emotional response to the image by reinterpreting the displayed scene, either by increasing their negative emotion to an originally neutral image (negate or up-regulate, arrow pointing up) or by reducing their emotional reaction to a negative picture, i.e., decreasing the negativity of the image (neutralize or down-regulate, arrow pointing down). In the view condition subjects were instructed to simply look at the picture attentively without trying to change their emotional responses toward it.

Each subject reappraised one of the neutral (“Reading” or “Working”) and one of the negative (“Crying” or “Violence”) categories, while the other two categories were passively viewed, i.e., kept their original affective values. Yet, only 9 of 15 images in every category were shown at this stage. The remaining images were only presented at the rating and AB parts in order to test whether the reappraisal effect can be generalized to non-regulated stimuli, which are similar, but not identical, to the reappraised ones. The allocation of scene-categories to the different regulation-strategy conditions and the selection of the seen image from each of the stimulus-pairs (see Stimuli) were counter-balanced between participants.

A trial of the regulation task began with a 4000 ms fixation cross followed by a 1000 ms induction phase in which an image was presented, followed by a 1500 ms instruction indicating the regulation-strategy (reappraise-negate, reappraise-neutralize, passive view). The image was then presented for an additional 6000 ms during which the subjects were asked to view the image applying the given instructions (Kanske et al., 2011; Schönfelder et al., 2013). In total, subjects performed one session that included a single presentation of 36 images (nine images for each of the four emotional value × regulation conditions).

Before the regulation task, a training session with 20 additional images (five for every scene-category) was conducted. The training procedure had two stages. At first, the subjects were presented with six view trials as well as six reappraisal trials with a sentence exemplifying the reappraisal strategy overlaid on the image (presentation duration was self-determined by subjects). For example, a neutral image of a girl reading a book was presented with the sentence “Reading a book on a bench at the hospital soon to discover she is sick with cancer” (reappraise-negate); a negative image of man being beaten up was coupled with the sentence “The police will soon intervene and the man will remain without any injury” (reappraise-neutralize). The second stage included four view and four reappraisal trials and the subjects were asked to verbally tell their reappraisal strategy to the experimenter.

#### Attentional blink task

**FIGURE 2:**
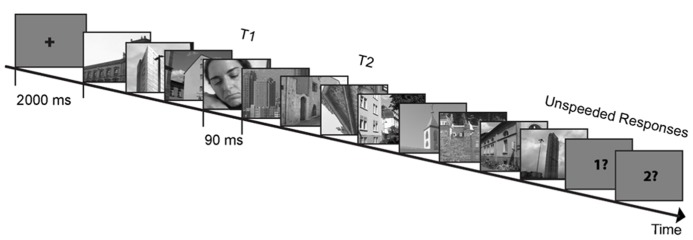
**Example trial and stimuli of the attentional blink paradigm.** In a rapid serial visual presentation, subjects were presented with two targets in a series of distractors. Subjects had to report the visibility (visible/unsure/invisible) of the first target (T1) and the rotation direction (left/right/don’t know) of the second target (T2; depicted stimuli are for illustrational purposes only).

In the following AB paradigm, subjects were presented with a RSVP that included two targets (T1 and T2) and a stream of distractors presented at ~11 Hz (presentation duration: 90 ms; **Figure 2**). They were informed about the stimuli identity and were instructed to pay attention to both targets and ignore distractors. Each trial began with a 1000 ms fixation cross. T1 appeared equally often at positions three to six of the visual stream. T2 appeared three positions after T1 in lag 3 trials (containing 10 distractors in each trial) or eight positions after T1 in lag 8 trials (containing 15 distractors in each trial to equalize number of distractors before T1 and after T2). Lag 3 and lag 8 trials occurred in different sessions. Lag 8 trials appeared in the last two AB sessions and were meant to be used as a control condition since T2 in this lag falls outside the AB critical temporal period (MacLean and Arnell, 2012). T1 was always a picture containing human beings, T2s were architectural or landscape images rotated 90° to the left or 90° to the right. Upright architectural or landscape images served as distractors. Subjects were instructed to make two unspeeded answers regarding the identity of the targets after each trial: (1) what was the visibility of humans in the T1 image [visible (i.e., human/s figures clearly appeared), unsure (i.e., not certain whether human/s were displayed), invisible (i.e., sure that no human/s figures appeared)]? (2) what was the orientation of the T2 image (90° left, 90° right, don’t know)? The next trial started after a response to the second question was made (**Figure 2**).

In total, subjects performed six sessions with 60 trials each. Each experimental session included all T1 and T2 stimuli. Since previous studies indicated a possible learning effect in the AB paradigm (Maki and Padmanabhan, 1994; Nakatani et al., 2012; Tang et al., 2013) and since lag 8 was included for control purposes only, lag 8 trials always appeared at sessions 5–6, i.e., in the last two sessions. Subjects performed eight training trials before the start of the AB task.

In all experimental stages (ratings, regulation task, AB), the order of the stimuli within a session was pseudo-randomized with no more than three consecutive repetitions of T1 valence value (negative/neutral), T1 regulation-strategy (neutralize/negate/passive view) and T2 rotation (only in the AB task). The time between the regulation task and the second rating was approximately 50 min and comprised the AB task (instructions, training, experimental task).

#### Questionnaires

At the end of the experiment, subjects reported their subjective feeling of effort [ranging from 1 (very easy) to nine (very effortful)] and success [ranging from 1 (very successful) to nine (very unsuccessful)] in regulating both negative (reappraise-neutralize) and neutral (reappraise-negate) emotions.

### APPARATUS

The experiment was presented using the Presentation software (Neurobehavioral Systems, Inc., Albany, CA, USA) running on a Windows computer. The TFT-LCD monitor (1440x900 resolution, 60 Hz refresh rate, Samsung SyncMaster 223BW) was positioned approximately 56 cm from the subjects’ eyes using a chin rest.

### STATISTICAL ANALYSES

The current study contained two major parts, which also guided the statistical analyses: (1) an emotion evaluation and regulation task and (2) an AB task. Subjects first rated all negative and neutral human images that were used throughout the experiment for their emotional valence and arousal. They then performed an ER experiment in which they either passively viewed the images, decreased their emotions to negative images (reappraise-neutralize) or increased their emotions to neutral images (reappraise-negate). Only 60% of all images appeared at that stage, enabling us to investigate if reappraisal can be generalized to similar, yet previously non-regulated stimuli. Subsequently, an AB task, in which the human-containing images served as the first target, was conducted. Finally, subjects rated the images a second time.

First, to evaluate the effect of cognitive reappraisal the two self-reported ratings (i.e., before and after reappraisal) were compared. For that purpose we conducted two separate 2 (rating-time: before vs. after reappraisal) × 2 (initial emotion: neutral vs. negative) × 2 (regulation-strategy applied on the scene-category: reappraise vs. passive view) × 2 (novelty: images seen at the regulation part vs. non-seen images) repeated measures ANOVAs (rANOVAs) of valence and arousal ratings. These ratings are frequently used as a measurement of the reappraisal effectiveness (Macnamara et al., 2009; Kanske et al., 2011; Schönfelder et al., 2013). Since all images were rated but not all were reappraised, the inclusion of the within-subject factor “novelty” enabled us to examine the generalization of the reappraisal effect to images of the same scene-category.

Second, we report the AB results with T2 detection accuracy contingent on the affective value and previous regulation-strategy [reappraise-negate, reappraise-neutralize, passive view, non-seen (i.e., did not appear at the regulation stage)] of the preceding T1 image. Using this paradigm attention modulation by reappraisal was investigated. First, we analyzed overall T1 detection rate over the different task blocks and for the different lags (lag 3 vs. lag 8) by calculating the percentage of visible choices out of the total number of trials. Possible habituation effects were evaluated using regression analysis and *t*-tests. To further evaluate ER effects on attentional processes we conducted a 2 (initial emotion: neutral vs. negative) × 2 (regulation-strategy applied on the scene-category: reappraise vs. passive view) × 2 (novelty: seen vs. non-seen images) rANOVA of T2 accuracy.

In case we observed significant interaction effects, we calculated *post hoc* rANOVAs where appropriate. For all analyses, effects were treated as significant when passing a significance threshold of *p *< 0.05 (two-tailed).

## RESULTS

### VALENCE AND AROUSAL RATINGS

Valence ratings showed a significant main effect of emotion [*F*(1,24) = 139.00, *p *< 0.001, partial η^2^ = 0.85] with negative images being rated as more unpleasant compared to neutral ones. The rating-time × emotion interaction [*F*(1,24) = 46.87, *p *< 0.001, partial η ^2^ = 0.66] demonstrated reduced difference between valence rating of negative vs. neutral images at the second rating task, after reappraisal. In addition, the 3-way interactions emotion × regulation-strategy × novelty [*F*(1,24) = 6.26, *p *= 0.20, partial η ^2^ = 0.21) and rating-time × emotion × regulation-strategy [*F*(1,24) = 7.44, *p *= 0.012, partial η ^2^ = 0.24] were significant. Most importantly, there was a 4-way interaction between rating-time × emotion × regulation-strategy × novelty [*F*(1,24) = 6.96, *p* = 0.014, partial η ^2^ = 0.22].

**FIGURE 3:**
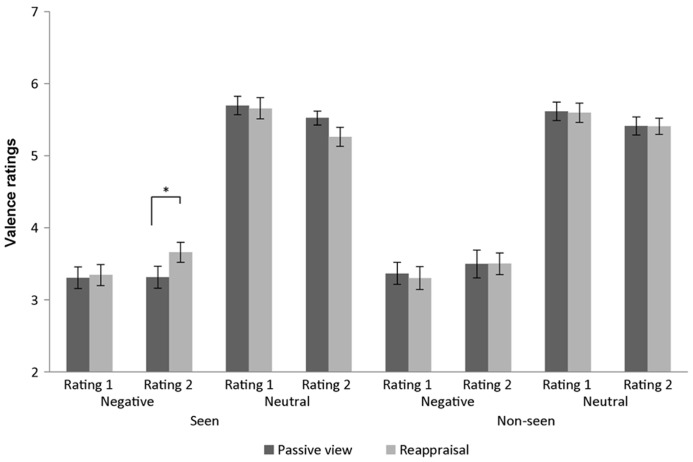
**Affective rating results.** Valence ratings from 1 (negative) to 9 (positive) obtained using the Self-Assessment Manikin scales in the 16 conditions (across subjects’ mean ± SEM). Data scaled for depiction. *p < 0.05.

To evaluate this 4-way interaction we conducted emotion × rating-time × regulation-strategy rANOVAs separately for seen and non-seen stimuli. Valence values for non-seen stimuli differed significantly only in terms of emotion main effect [*F*(1,24) = 155.55, *p *< 0.001, partial η ^2^ = 0.87] and emotion × rating-time interaction [*F*(1,24) = 21.70, *p *< 0.001, partial η ^2^ = 0.47] with increased valence values (toward more neutral values) for neutral images [*t*(24) = 2.36, *p *= 0.026, mean difference = 0.196] and decreased negativity for negative images [*t*(24) = -2.26, *p *= 0.033, mean difference = -0.166] when comparing the second to the first rating task. Valence ratings for seen stimuli revealed a main effect of emotion [*F*(1,24) = 117.74,*p*< 0.001, partial η ^2^ = 0.83], rating-time × emotion interaction [*F*(1,24) = 22.72, *p *< 0.001, partial η ^2^ = 0.49], as well as a rating-time × emotion × regulation-strategy interaction [*F*(1,24) = 11.67, *p *= 0.002, partial η ^2^ = 0.33]. Therefore, two emotion × regulation-strategy rANOVAs were further calculated for the two rating tasks. While for both rating tasks, we found an emotion main effect [first rating: *F*(1,24) = 124.31, *p *< 0.001, partial η ^2^ = 0.84; second rating: *F*(1,24) = 98.59, *p *< 0.001, partial η ^2^ = 0.80], the emotion × regulation-strategy interaction was only significant at the second rating task [*F*(1,24) = 10.11, *p *= 0.004, partial η ^2^ = 0.30], i.e., after the reappraisal procedure. Indeed, *post hoc* *t*-tests revealed that negative images reappraised as neutral were more pleasant than negative images that were previously passively viewed and thus retain their affective value [*t*(24) = -3.89, *p *= 0.001, mean difference = -0.344]. On the other hand, we observed a non-significant difference in valence ratings for neutral pictures that were reappraised to be more negative vs. passively viewed neutral images [*t*(24) = 1.68, *p *= 0.105; mean difference = 0.259; **Figure 3**). There was no other significant effect.

The arousal ratings were less affected by the experimental manipulation. The 2 × 2 × 2 × 2 rANOVA of the arousal ratings revealed emotion [*i*(1,24) = 55.25, *p *< 0.001, partial η ^2^ = 0.70] and rating-time [*F*(1,24) = 5.49, *p *= 0.028, partial η = 0.19] main effects. There was a regulation-strategy × novelty interaction [*F*(1,24) = 10.85, *p *= 0.003, partial η ^2^ = 0.31] due to higher arousal values for non-seen stimuli compared to seen stimuli from the reappraised categories [*t*(24) = 3.26, *p *= 0.003, mean difference = -0.181]. In addition, the interactions emotion × rating-time [*F*(1,24) = 20.60, *p *< 0.001, partial η ^2^ = 0.46] and emotion × rating-time × regulation-strategy [*F*(1,24) = 5.57, *p *= 0.027, partial η ^2^ = 0.19] were significant. Supplementary emotion × regulation-strategy rANOVAs separately for the two ratings were in turn conducted. The rANOVAs demonstrated an emotion main effect for both the first [*F*(1,24) = 66.22, *p *< 0.001, partial η ^2^ = 0.73] and the second ratings [*F*(1,24) = 41.89, *p *< 0.001, partial η ^2^ = 0.64], indicating higher arousal values for negative images. At the second rating there was a trend for 2-way interaction [*F*(1,24) = 3.34, *p *= 0.080, partial η ^2^ = 0.12]. Neutral images that were reappraised to be negative increased their arousal values at the second rating task, as shown by *post hoc* tests [*t*(24) = 2.82, *p *= 0.010, mean difference = 0.181]. No significant difference in arousal values was found for negative images [*t*(24) = -1.02, *p *= 0.319, mean difference = -0.184] due to high variability (SD = 0.90). No other effect was significant.

### ATTENTIONAL BLINK

Overall, subjects reported the humans to be visible in the T1 targets in 97.88 ± 2.17% (mean ± SD) and 97.70 ± 2.49% of lag 3 and lag 8 trials, respectively. Since the study investigated the modulation of T2 by the valence and regulation history of T1, it is important to confirm that T1 images were indeed perceived. In accordance with the conventional AB analysis, T2 identification rate was contingent on T1 visibility, i.e., only trials in which T1 was reported to be visible (i.e., seen) were analyzed. In order to detect any training effect, we next analyzed the T2 detection accuracy separately in each of the six sessions. The overall T2 detection accuracy was higher for lag 8 (84.21 ± 11.64%) compared to lag 3 (80.02 ± 13.18%) trials [*t*(24) = 2.94, *p *= 0.007]. However, regardless of lag (lag 3 in sessions 1–4, lag 8 in sessions 5–6) there was a gradual increase in T2 detection rate over time [β = 0.95, *t*(4) = 6.34, *p *= 0.003; *R*^2^ = 0.91, *F*(1,4) = 40.25, *p *= 0.003]. Upon pair-wise comparisons of consecutive sessions, there was a significant increase in the mean accuracy during the task only between the second (78.5 ± 14.04%) and third (82.5 ± 14.82%) sessions [*t*(24) = -3.03, *p *= 0.006]. Therefore, performance improved with increasing exposure to the stimulus-material in the AB task, as previously suggested (Maki and Padmanabhan, 1994; Nakatani et al., 2012; Tang et al., 2013), with a particularly steep increase after the second session in our study. The training effect, i.e., reduced mistakes with the progress of the experiment, may be confounded with the influence of T1’s affective valence on the error/accuracy pattern. Since this is the variable we are interested in, it is important to make sure that the response pattern is not contaminated due to the training. Thus, in order to reliably detect the reappraisal effect we only included the first and second AB sessions in the analysis.

**FIGURE 4:**
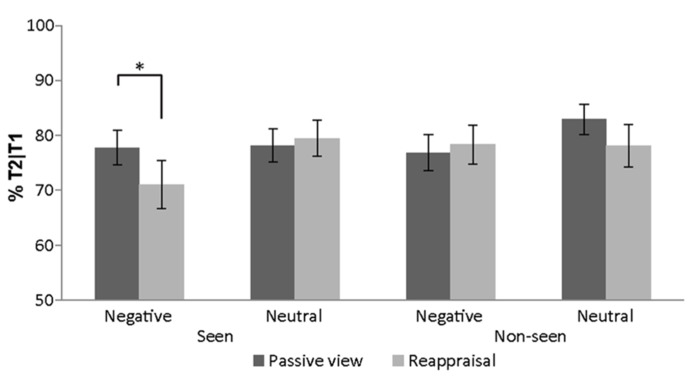
**Attentional blink results.** Detection accuracy of T2 given T1 was visible, as a function of T1 affect and regulation-strategy (across subjects’ mean ± SEM). *p < 0.05.

The rANOVA (emotion × regulation-strategy × novelty) of T2 accuracy in the first two sessions revealed a main effect of emotion [*F*(1,24) = 5.54, *p *= 0.027, partial η ^2^ = 0.19] and a 3-way interaction [*F*(1,24) = 7.48, *p *= 0.012, partial η ^2^ = 0.24]. Two emotion × regulation-strategy rANOVAs where then conducted, separately for seen and non-seen stimuli. For seen stimuli there was a main effect for emotion [*F*(1,24) = 4.27, *p *= 0.0497, partial η ^2^ = 0.15] which was driven by a significant emotion × regulation-strategy interaction [*F*(1,24) = 5.68, *p *= 0.025, partial η ^2^ = 0.19]. *Post hoc* *t*-tests revealed higher T2 detection accuracy after negative images that were simply viewed compared to negative images that were previously reappraised as neutral [*t*(24) = 2.26, *p *= 0.033, mean difference = 6.7%]. No such difference was found for neutral images [*t*(24) = -0.59, *p *= 0.563, mean difference = -1.3%; **Figure 4**]. For non-seen stimuli, on the other hand, we observed no significant main or interaction effects.

For completion we also report the rANOVA (emotion × regulation-strategy × novelty) of T2 accuracy for the last two lag 3 sessions (i.e., sessions 3 and 4). Only a significant regulation-strategy main effect was found [*F*(1,24) = 6.02, *p *= 0.022, partial η ^2^ = 0.20], with higher performance for non-regulated images. The increased accuracy between the second and the third sessions was therefore probably caused by the reduced influence of T1s on the performance due to stimulus repetition.

### QUESTIONNAIRES

Subjects rated their feeling of success and difficulty (effort) in regulating their emotions (Ochsner et al., 2004). Overall, subjects reported to exert more effort [*t*(24) = 2.54, *p *= 0.018] and being less successful [*t*(24) = 1.97, *p *= 0.060] when reappraising neutral images as negative (reappraise-negate) compared to reappraising negative images as neutral (reappraise-neutralize). No correlation was found between the subjective ratings and the performance in the AB task.

## DISCUSSION

The current study investigated modulation of attentional processes through cognitive ER as well as the generalization of these effects in healthy individuals. More precisely, participants passively viewed or reappraised neutral and negative images, which later appeared as T1 in an AB task. By analyzing T2 detection rate as a function of initial image valence and regulation-strategy we sought to determine the influence of ER on attentional processes. Indeed, our results indicate that cognitive reappraisal affected AB performance as shown by decreased accuracy rates for negative images that were reappraised as neutral compared to negative images which were passively viewed. However, this effect was not generalized across stimuli that were similar in their semantic meaning but have not been actively reappraised before.

The results of the present study are not only evidence for a modulatory effect of reappraisal on attention but also pave the way for developing more implicit behavioral measures of ER success, which is, to date, mostly assessed by means of self-report measures. Such measurements might, however, be subjected to participants’ predictions regarding the experimental aim and their individual ability to reflect upon their emotions (Nielsen and Kaszniak, 2007). Having an independent behavioral task in addition to the ratings allows better characterization of reappraisal’s influence on emotion.

When negative images served as T1, performance in the AB task was modulated by the reappraisal condition. Negative images that have been earlier reappraised as neutral reduced T2 detection rate compared to negative images which were only viewed. In other words, less emotional stimuli hindered more the performance. Indeed, actively decreasing the emotional value for negative images was successful as seen from the subjects’ explicit ratings. Compared to passively viewed negative images, reappraised ones were rated as less negative in the second rating task after the regulation. The reported AB results seem at first to be at odds with previous reports of attention capture by affective stimuli (Anderson and Phelps, 2001; Keil and Ihssen, 2004; Anderson, 2005; Arnell et al., 2007; Most et al., 2007). For example, when subjects detected a single target in a RSVP of distractors, detection was reduced when one of the distracters was an arousing vs. neutral stimulus (Arnell et al., 2007; Most et al., 2007). While those previous studies placed a special emphasis on arousal values, in the present experiment arousal values were less influenced by the reappraisal manipulation compared to the valence ones, as indicated by the subjective ratings. Additionally, our AB results mainly reflect the reappraisal manipulation. The initial emotional value of passively viewed images did not greatly affect AB performance since passively viewed negative compared to neutral T1 stimuli did not substantially decrease T2 detection rate (**Figure 4**). We suggest that this may be related to a habituation process over repeated presentation of the images during the first rating, reappraisal and AB tasks, which might have reduced the impact of their affective content.

Reappraisal is a dynamic process (Gross and Thompson, 2007; Kalisch, 2009) which requires working memory and uses limited cognitive and attentional resources (Wegner et al., 1993; Schmeichel et al., 2008; McRae et al., 2012b; Ortner and de Koning, 2013; Ortner et al., 2013). In fact, higher working memory capacities contributes to higher reappraisal success (Schmeichel et al., 2008) and training subjects in an emotional working memory task increases reappraisal success after the training (Schweizer et al., 2013). Seeing the pictures again in a different context (i.e., in the AB task) might have triggered retrieval of the images’ new meaning from long term memory (Macnamara et al., 2011), a process described earlier as maintenance (Kalisch, 2009). In other words, it is possible that the images were first appraised according to their original content and that their affective value was subsequently modified based on the prior reappraisal. Such a maintenance or updating process might happen implicitly (which is quite probable) or explicitly, however, this distinction cannot be made from the current experimental task. This maintenance or updating stage, which occurs if T1 was previously reappraised (compared to merely viewed), requires elevated processing time. Due to the capacity-limited resources (either attentional or information-processing process such as consolidation; Marois and Ivanoff, 2005; Martens and Wyble, 2010), longer T1 processing reduces the resources available for T2 computation. This implies that reappraisal (at least when it is performed once per stimulus) does not create a permanent affective change and that it is a relatively slow process that depends on cognitive resource. While more studies are required to validate this proposed model it agrees with recent observations of temporally and anatomically distinct neuronal activity during reappraisal, where late activity is assumed to reflect the maintenance phase (Kalisch, 2009; Paret et al., 2011).

An alternative account to our results is related to the extended processing of stimuli. Accordingly, reappraised images capture more attention simply because they have undergone extensive processing earlier. The observed decline in AB performance is thus independent from the reappraised affective value of the images. While we cannot fully exclude this explanation, we believe it cannot completely account for the current results. As revealed by subjects’ effort ratings at the end of the experiment, reappraisal of neutral images was rated as more effortful than negative images and should therefore have been more extensively processed according to this explanation. However, reappraised neutral images did not alter AB performance in comparison to only viewed neutral images.

Only few studies have previously investigated the enduring influence of reappraisal (Walter et al., 2009; Macnamara et al., 2011; Thiruchselvam et al., 2011; Ortner et al., 2013). While in the current study upon re-exposure (rating 2) the valence ratings of negative images were affected by the reappraisal history, Thiruchselvam et al. (2011) did not find such an effect when subjects viewed the images 30 min after regulation. In their study, subjects first rated each image immediately after it was regulated or viewed, possibly enhancing the difference in self-reports between the two conditions, and at the same time reducing this difference in the second rating during the re-exposure when all the images were simply viewed. In the current experiment, the two rating tasks were identical avoiding differences due to modification between the tasks. Reappraisal was also shown previously to exhibit long lasting neuronal effects. Specifically, amygdala activity, which is largely thought to reflect processing of unpleasant affects (Costafreda et al., 2008; Sergerie et al., 2008), was reduced upon re-exposure to reappraised negative images (Walter et al., 2009). Similarly, event-related potential (ERP) amplitudes were reduced during re-exposure to negative, previously reappraised, images, especially at late time windows (Macnamara et al., 2011; Thiruchselvam et al., 2011). Collectively, this and previous studies suggest that reappraisal has a long lasting effect. Indeed, when people are free to choose which regulation-strategy to employ, they often choose reappraisal if multiple exposures to the stimuli are expected (Sheppes et al., 2012; Sheppes and Levin, 2013).

The influence of reappraisal on attention and emotional-state might depend on the extent of training. If the reappraisal strategy for a specific stimulus is extensively trained by re-exposure to that stimulus, the affective change might become more accessible to memory and capture fewer resources upon retrieval. Along those lines, a recent study showed a continued reduction in negative affect due to longitudinal reappraisal training (Denny and Ochsner, 2013). In addition, the enduring and accessibility of the cognitive change may largely depend on the specific regulation-strategies employed (e.g., reappraisal, mindfulness). In our experiment subjects were instructed to use situation-focused reappraisal (i.e., to change the meaning of the situation) and therefore upon re-exposure depended on the exact interpretation they have applied to the image. Self-focused reappraisal, on the other hand, involves distancing oneself from the situation and might be implemented in a more permanent manner after training (Denny and Ochsner, 2013). In other words, it might require less resources to access a distanced vs. reinterpreted meaning of a scene. Indeed, both types of reappraisal recruit different subparts of the prefrontal cortex (Ochsner et al., 2004).

### LIMITATIONS AND FUTURE DIRECTIONS

If reappraisal requires cognitive resources we would expect all regulated images to affect attention, regardless of their initial affective value. No such effect was found for the neutral images. In other words, neutral stimuli that were reappraised to be negative did not hinder T2 detection more than non-regulated neutral stimuli. The valence ratings provide an explanation to those results, with no significant difference between self-reports of the two neutral categories at the second rating task. In addition, the absolute ratings of the neutral pictures were slightly more positive than the normative ratings, possibly since subjects rated the neutral images in comparison to the negative ones. The current rating results differ from previous studies by Macnamara et al. (2009, 2011) in which the ratings of neutral images were more influenced by the reappraisal manipulation. This can be attributed to the experimental design itself (subjects heard a specific auditory description prior to every image in their studies) or due to differences in the subject population. In fact, the subjects of the current study indicated that they exerted more effort and were less successful in the regulation condition where neutral images had to be reappraised to be more negative (likely because they were perceived as slightly positive). It is possible that this condition did not create a successful cognitive change among our subjects, and hence did not significantly affect the performance in the AB task. Specifically, attributing negative meaning to neutral stimuli might be difficult to healthy individuals, as this form of reappraisal does not serve an adaptive function in daily life. It would be important to further investigate the observed discrepancy between negative and neutral stimuli in the regulation-strategy.

We also found no indication for generalization. Images that were not reappraised, but were taken from the same scene-category as the reappraised images, did not alter AB performance or the valence ratings. Further, the non-seen images were rated as more arousing. Although not much can be proven by non-significant results, the lack of generalization may indicate that reappraisal has to be directed on a stimulus-by-stimulus basis for successful affective change. Alternatively, the generalization of reappraisal effect might depend on the degree of similarity between the familiar and novel stimuli. While our stimuli stemmed from the same scene-category, they might have still been too diverse to allow generalization. This will have to be determined in future research.

Finally, although the second rating task did not occur directly after the reappraisal phase, the experimental delay in the current experiment (i.e., the AB task) was not much longer than in previous experiments. It is necessary to test longer delays such as hours and even weeks in order to capture the exact time-frame of reappraisal’s lasting effect. This is particularly important from a clinical perspective since reappraisal has a long-standing tradition in cognitive-behavioral psychotherapy.

## CONCLUSION

Our results suggest that reappraisal can modulate attentional processes. Specifically, performance in the AB paradigm was attenuated due to the reappraisal manipulation of negative images, i.e., the reinterpretation of negative stimuli as more neutral. Whereas T2 detection rate did not differ between only viewed negative and neutral images, it was decreased when negative images that were previously reappraised as neutral preceded T2. Presumably, reappraised negative images captured more resources than those that were only viewed, possibly due to the dynamic nature of the cognitive reappraisal process. Furthermore, reappraised negative stimuli were rated as less negative following the AB task, pointing to a lasting effect of reappraisal. Future research should examine other task contexts and manipulate the duration between the regulation and the evaluative tasks to elucidate on these issues. Most importantly, future studies should investigate various stimuli to determine if the reappraisal effect can be generalized to novel situations, investigate the effect of long reappraisal training periods and further validate our conclusion.

## Conflict of Interest Statement

The authors declare that the research was conducted in the absence of any commercial or financial relationships that could be construed as a potential conflict of interest.

## Footnotes

1 IAPS (4-digits), EmoPicS (3-digits) and NAPS (faces/people prefix) identification numbers. “*Reading”*: 140, 141, 142, 145, 165, 176, 178, 181, 183, 184, 201, 2102, 2374, 2377, People_163_v; “*Working”*: 119, 120, 121, 123, 128, 130, 132, 135, 136, 138, 2026, 2393, 2579; Faces_229_v, Faces_310_v; “*Crying”*: 208, 214, 218, 219, 220, 222, 223, 224, 225, 226, 2456, 9220, 2900, Faces_283_h, People_001_h; “*Injury”*: 238, 245, 246, 247, 249, 2345.1, 3017, 3051, 3181, 3550, 9250, 9265, Faces_294_h, Faces_174_h,People_010_h.
